# Back to the future: revisiting MAS as a tool for modern plant breeding

**DOI:** 10.1007/s00122-018-3266-4

**Published:** 2018-12-17

**Authors:** Joshua N. Cobb, Partha S. Biswas, J. Damien Platten

**Affiliations:** 1 International Rice Research Institute, National Road, Los Banos, Laguna Philippines; 2 Bangladesh Rice Research Institute, Gazipur, 1701 Bangladesh

## Abstract

**Key message:**

New models for integration of major gene MAS with modern breeding approaches stand to greatly enhance the reliability and efficiency of breeding, facilitating the leveraging of traditional genetic diversity.

**Abstract:**

Genetic diversity is well recognised as contributing essential variation to crop breeding processes, and marker-assisted selection is cited as the primary tool to bring this diversity into breeding programs without the associated genetic drag from otherwise poor-quality genomes of donor varieties. However, implementation of marker-assisted selection techniques remains a challenge in many breeding programs worldwide. Many factors contribute to this lack of adoption, such as uncertainty in how to integrate MAS with traditional breeding processes, lack of confidence in MAS as a tool, and the expense of the process. However, developments in genomics tools, locus validation techniques, and new models for how to utilise QTLs in breeding programs stand to address these issues. Marker-assisted forward breeding needs to be enabled through the identification of robust QTLs, the design of reliable marker systems to select for these QTLs, and the delivery of these QTLs into elite genomic backgrounds to enable their use without associated genetic drag. To enhance the adoption and effectiveness of MAS, rice is used as an example of how to integrate new developments and processes into a coherent, efficient strategy for utilising genetic variation. When processes are instituted to address these issues, new genes can be rolled out into a breeding program rapidly and completely with a minimum of expense.

## Introduction

The advent of the Green Revolution in the 1960s brought a step change in potential yields for rice and wheat and is credited with avoiding severe food crises (Pingali 2012). Since this time, there has been a constant expectation that plant breeding efforts will be able to sustain gains in yield, ironically against a background of decreasing funding (American Society of Agronomy 2018). At the same time, the new intensive cropping systems promoted by the Green Revolution resulted in increased pressure from pests and diseases while farm areas continue to push into more marginal land (Tilman et al. 2002). To meet these challenges, breeders and geneticists have been very successful in identifying sources of novel genetics from pre-Green Revolution landraces with the intention of bringing various biotic and abiotic stress tolerances into the high-yielding semi-dwarf backgrounds prevalent in farmers’ fields today (see reviews by Gilliham et al. 2017; Bailey-Serres et al. 2010).

As molecular biology grew as a discipline, interest naturally intensified in identifying heritable variation of value, particularly by characterising and annotating the underlying genes that comprise the genetic architecture of relevant traits. In addition to the academic value of this exercise, the possibility of imposing selection directly on these genes/QTLs using molecular techniques has been recognised for decades and is often referred to today as ‘molecular breeding’ (Moose and Mumm 2008). Compared to phenotypic selection, molecular breeding methods offer several key advantages: they are typically faster, can be done on seedling-stage material, permit the enrichment of populations with heterozygous individuals through codominance, can be substantially cheaper, and have a heritability of essentially 1.0 (Collard and MacKill 2008).

The concept of MAS has been used extensively as justification to identify and clone hundreds of genes across many species (Song et al. 1995; Qu et al. 2006; Ji et al. 2016). Rice in particular boasts dozens of cloned genes with significant phenotypic effects and serves as a useful case study to understand both the potential value of marker-assisted selection and its barriers to deployment. The rich genetic variation amenable to MAS in rice is a function of the partitioning of rice genetic diversity (Govindaraj et al. 2015; The 3000 Rice Genomes Project 2014) and its adoption as the first model species in monocots and subsequent worldwide effort to publish its genome sequence (IRGSP 2005). For example, the Q-TARO database currently contains 114 cloned genes with natural variants affecting various traits (Yonemaru et al. 2010), and the number of identified QTLs is many times this value.

## Impact of MAS

**Fig. 1:**
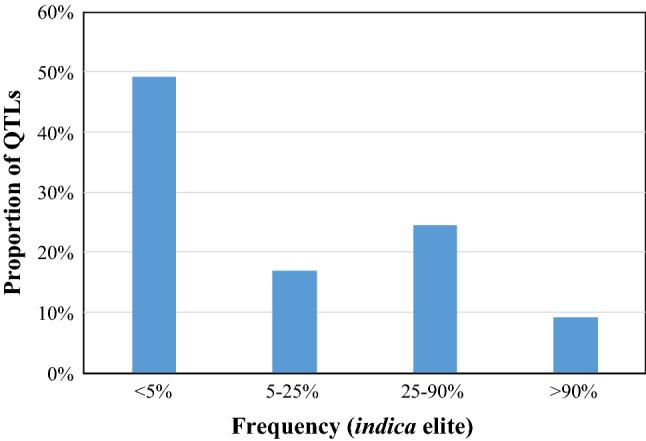
Frequency of key QTLs in elite *indica* breeding material. Over sixty well-validated genes and QTLs controlling a range of disease resistance, grain quality, and abiotic stress traits were examined in over 75 elite *indica* varieties based on whole-genome sequencing data. Almost half of these genes were absent in elite material; another 17% were very are. Only about 25% are available for easy selection in forward breeding

Despite the huge success in identifying QTLs controlling a wide variety of traits in different species and the identification of the functional variants underlying these QTLs, the success of marker-assisted selection for major genes in large public breeding programs has been limited (Collard and MacKill 2008). Among elite indica rice breeding germplasm, a survey of over 60 such genes and QTLs shows that almost half are not found in elite programs at all, and another 17% are very rare (Fig. 1). Discounting the 9% that were essentially already fixed across *indica* germplasm, only 25% of genes amenable to marker-assisted selection are available to breeders.

**Fig. 2:**
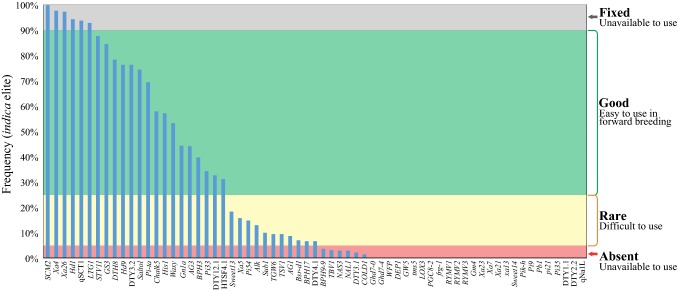
Breakdown of frequency of key genes in elite *indica* germplasm. The frequency of high-value genes controlling a range of important traits was assessed on genome sequences of 75 elite *indica* breeding lines and varieties from a range of rice breeding programs around the world. The majority of genes, including many of the most effective disease resistance loci, were absent from elite material

The cause behind this limited deployment of major genes in public rice breeding programs has been debated many times, and useful reviews of potential factors were put forward (William et al. 2007; Collard and MacKill 2008). Lack of awareness of the genetic variants or their value seems unlikely. As a case in point, *Xa21* and *Pi9*, two of the most effective, broad-spectrum resistance genes against bacterial blight and blast disease, respectively, were both identified more than 30 years ago (Amante-Bordeos et al. 1992; Song et al. 1995). Parents possessing these genes have been used extensively in crossing programs—86% of IRRI’s irrigated rice breeding program is related to IR24, a consequence of extensive crossing with IRBB near-isogenic lines (data not shown)—yet still their value is yet to be realised in modern varieties. Furthermore, marker-assisted utilisation and pyramiding of various genes have been reported by several groups (e.g. Singh et al. 2001; Kumar et al. 2013; Yasuda et al. 2015), yet these products do not appear to have made impact as varieties nor have their progeny increased in frequency relative to susceptible genotypes (Fig. 2).

Comparison of the frequency of various genes in elite *indica* germplasm may give a clue as to the cause of this absence. When examining genes within the major disease resistance categories (blast and bacterial blight), it is evident that frequencies of different genes fall into roughly two groups; a small number are at reasonable frequencies (> 25%, e.g. *Xa4*, *Xa26, Pi*-*ta, BPH32*), while nearly all the remainder are absent (Fig. 2). This may be an indication that certain genes have found their way into breeding programs, perhaps through a high basal frequency among founders or through crossing with donor lines followed by phenotypic selection. These are now segregating to varying extents, but in doing so are potentially epistatic with new loci and interfere with the ability to reliably select for new loci phenotypically. If this were the case, introduction of new genes via phenotypic selection would be haphazard and largely unsuccessful, as is observed.

The question remains then, if phenotypic selection for major genes is not effectively increasing the frequency of new useful loci due to epistatic effects with the few loci already at high frequency, why not use molecular markers to increase their prominence deterministically? Understanding the psychology and behavioural economics of plant breeders is a very interesting and under-studied area of research (Coors 2006; Lenaerts et al. 2018); however, one might hypothesise that lack of confidence in marker-assisted selection could play a role. Not to be confused with a lack of understanding of the inheritance patterns that underlie MAS or a lack of knowledge about the QTL, we suggest that this potential lack of confidence more likely stems from a very pragmatic lack of phenotypic improvement observed when MAS is applied in breeding populations. MAS, as a method of indirect selection, depends on the association of phenotypic variation with genetic variation at a specific locus as surveyed by a particular marker (i.e. the trait ↔ marker correlation). But this itself actually comprises two underlying correlations. Obviously, the phenotype of interest needs to be unequivocally associated with the genomic region (i.e. the trait ↔ QTL correlation), determination of which is the focus of QTL mapping algorithms (Miles and Wayne 2008). However, even if a QTL explains 100% of the phenotypic variance for a trait of interest in a gene pool, the QTL still needs to be unequivocally associated with the marker used to impose selection for it (i.e. the QTL ↔ marker correlation). If either of these correlations (trait ↔ QTL or QTL ↔ marker) is poor, the trait ↔ marker association will not reliably improve the phenotype of interest, and so breeders may not only lose confidence in the value of a particular locus, but potentially in MAS itself as an effective selection method.

Indeed, much of the MAS that could have taken place for major genes in rice breeding populations also would have needed to occur at a time characterised by poor access to high-throughput marker systems (William et al. 2007; Rasheed et al. 2017; Steele et al. 2018). However, in the last decade, cheap, efficient, and pipelined markers systems have been made available (Steele et al. 2018). These advanced marker systems streamline the data collection process, but of course do not address the underlying challenge of ensuring that the information content of the markers is relevant and useful to a breeding program. This will involve addressing both correlations underlying MAS as an indirect selection strategy (trait ↔ QTL and QTL ↔ marker).

This work aims to outline a framework for ensuring these correlations are properly addressed in modern molecular breeding programs and provide some insight into how MAS can be integrated into a modern genomics-enabled breeding strategy. To this end, we will highlight ideas and methods under development at the International Rice Research Institute and partner organisations to improve this situation through improved tools for MAS (i.e. reliable markers and QTLs) and improved strategies for integrating these tools into the breeding process effectively. While genomic selection could be considered a form of marker-assisted selection, it is sufficiently distinct that integrating it into the breeding process warrants a separate discussion and is out of scope for this treatment of the topic.

## Ensuring effective MAS

It is not often appreciated that marker-assisted selection is fundamentally an indirect selection method. As mentioned, it relies on two correlations for it to be effective:

$$ {\text{Trait}}\;\left( {\text{phenotype}} \right)\; \leftrightarrow \;{\text{Gene/QTL}} $$

$$ {\text{Gene}}/{\text{QTL}}\; \leftrightarrow \;{\text{Marker}} $$

The importance of the first correlation has been recognised previously (e.g. Collard and MacKill 2008), but strategies to improve confidence in QTLs for traits still need to be developed and standardised across the discipline. Strangely, the second correlation has been almost ignored in the literature to date.

### How can we have confidence in a locus?

In defining what factors must be assessed when determining the reliability of the trait ↔ QTL correlation, it is essential to ensure the exercise is objective oriented. If the QTL is intended to be used by breeders to improve the level of some target trait in their breeding program, it must have certain characteristics:

1. It must provide measurable improvement in field performance.
2. It must be effective across the genetic diversity present in the breeding program(s).
3. Its effect size (balanced by the trait’s priority in the breeding program) must be sufficient to justify the expense of isolation, development, and deployment of the locus.

Understanding these criteria then influences how such activities as mapping of loci are conducted. For example, many QTL mapping studies have been conducted for salinity tolerance in rice, nearly all of which focus on sodium ion (Na^+^) content in the leaf (Lee et al. 2007; Ammar et al. 2009; Islam et al. 2011; Horie et al. 2012; Rahman et al. 2017). This makes physiological sense as sodium content is significantly correlated with visual injury score, yet QTLs for Na^+^ content have not made large impacts in improving salinity tolerance. This apparent conundrum illustrates the pitfalls of relying on correlated traits. Sodium content shows a highly significant correlation of up to 65% with visual injury (Platten et al. 2013). However, even if a modest-effect QTL correlated with Na^+^ content explained 20% of the variation in a given mapping population, this would only equate to (0.2 × 0.65) = 13% improvement in visual injury symptoms, too small to be apparent to a casual observer, especially in the field. It should also be noted that this is a 13% improvement relative to the phenotypic range observed in that specific mapping population; it does not reflect the absolute level of improvement, which may be much smaller when averaged across multiple genetic backgrounds. To avoid such situations, geneticists, physiologists, and pre-breeders need to consider more fully several components of the gene discovery process to ensure relevance to modern breeding programs.

#### Phenotypic considerations

The importance of phenotyping is not contentious, and significant literature has been written on different technologies and strategies for its use in breeding (e.g. Araus et al. 2018). It is not the intention of this article to recap this literature, but rather to highlight one salient point for QTL mapping exercises: phenotyping strategies must target the *actual traits* under selection to be useful for breeding purposes. While controlled environments, convenient phenotypic proxies, and component traits make for convenient mapping targets because they offer high heritability and often reduced cost, they often do not translate into meaningful selection targets for a breeding program unless specifically correlated with the trait of interest. For example, for salinity tolerance, a QTL controlling 20% of the variation in visual injury symptoms in the target environment under the stress of interest would be far preferable and more reliable than one controlling 20% of the variation in leaf sodium content. Mapping of QTLs for these direct traits—even when these traits have been difficult to map—has been very effective for identifying QTLs controlling yield under reproductive-stage drought stress in rice (Bernier et al. 2007).

Of course, phenotyping direct selection targets in the field is far more challenging and expensive than in the glasshouse. Mapping directly under relevant conditions creates scenarios with more spatial and temporal variation that reduce heritability and lead to decreased genetic signal. However, the benefits are that any detectable genetic signal that is observed is directly relevant to improvements that may be expected in breeding programs. The effect of environmental noise in field phenotyping protocols can be reduced through well-replicated field designs, better sampling of the targeted population of environments, and using genetically structured mapping populations such as RILs or CSSLs (Doi et al. 1997; Kubo et al. 2002). There are still, many situations where phenotyping an entire mapping population based on field observations is impractical or impossible. In these situations, evaluating populations under controlled conditions in a glasshouse or other artificial environment can successfully generate hypotheses about specific genetic loci. Once developed, these hypotheses can be tested and validated under field conditions following the creation of near-isogenic lines such as those produced from QTL deployment (discussed below).

Under such conditions, it is entirely possible that there will be no single genetic locus identified with a major contribution to a complex trait. In this situation, it is more likely that an additive polygenic or omnigenic model (Boyle et al. 2017) applies and breeders will make more progress using phenotypic or genomic selection to accumulate favourable alleles through successive cycles of breeding. See Cobb et al. (2013) for a more extensive review of phenotyping considerations for crop improvement in the context of forward breeding.

#### Genomic considerations

Even the best-designed and relevant phenotyping strategy will still be ineffective if the resulting QTLs do not work in the genomic backgrounds of the target breeding programs. This appears to be a major weakness of many QTL mapping and gene cloning studies; effectiveness of the QTL is often not demonstrated at all (only mapping results shown), or only demonstrated in a narrow range of genomic backgrounds that have limited relevance to contemporary breeding programs. Using salinity tolerance in rice as an example again, mapping studies have focused on populations utilising varieties such as IR29 (Rahman et al. 2017; Tiwari et al. 2016; Bimpong et al. 2014), Azucena (Zheng et al. 2003; Gomez et al. 2006; Khowaja et al. 2009) and Nipponbare (Wissuwa et al. 2002; Zhou et al. 2016) as recipient parents. These were chosen due to their extremely low level of salinity tolerance—thus maximising the phenotypic variation present in the resulting populations and hence the statistical power to detect QTLs. Unfortunately, these three lines are very old varieties or even *japonica* landraces that would be inappropriate for use in an elite *indica* breeding program, raising the question of whether or not QTLs identified in these backgrounds would even be effective in modern breeding programs. The observation that the majority of elite *indica* lines already possess one of the favourable alleles of *Saltol* (Fig. 2, Platten et al. 2013) is a convenient illustration of a QTL that may be perfectly functional, but only of value in backgrounds not relevant to breeding efforts.

In addition, since the chosen recipient varieties are typically more sensitive than most breeding lines (in order to maximise the probability of finding a QTL), there is the real danger that any QTLs identified explain not why the donor line is of particular value, but rather why these recipients have poor performance. The distinction between these points may be subtle, but is important. If the QTL explains why the donor performs well for the desired trait, the desirable QTL haplotype is likely to be rare or absent in the elite breeding material—otherwise most breeding material would also have a high trait value. On the other hand, if the QTL explains why the recipient (e.g. IR29) has low performance, the undesirable QTL haplotype is likely to be rather specific to that variety. Thus, the desirable QTL haplotype may well be frequent in the breeding pool (i.e. it would likely also be identified in a population from an average elite line crossed with that sensitive recipient) and so of little value in improving the trait for most elite lines. To avoid this situation, recipients should not be chosen specifically for their low performance for the target trait. More relevant results would be ensured by choosing elite recipients with average performance.

**Fig. 3:**
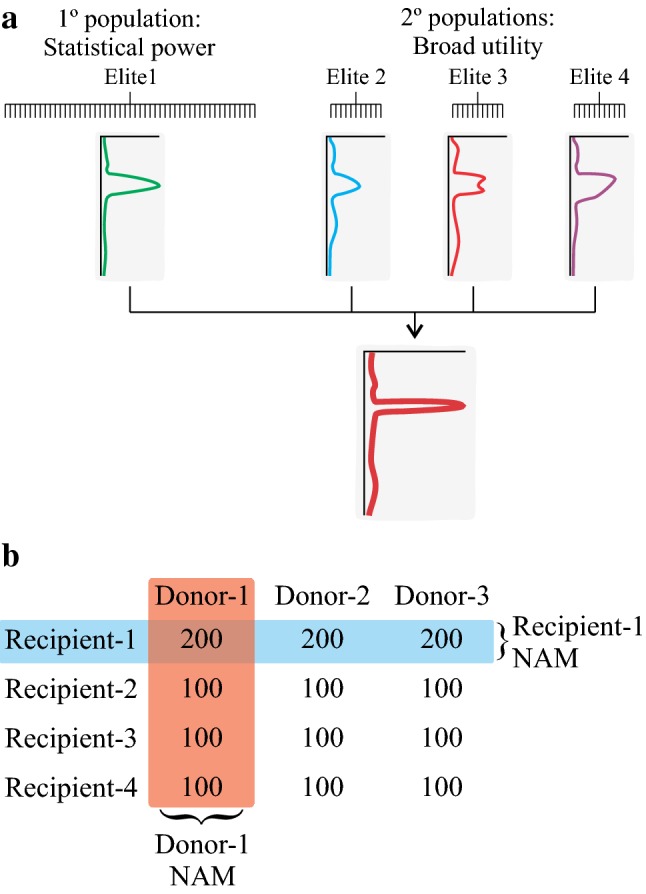
Basic strategy for mapping of QTLs through a half-diallel crossing. **a** For each donor identified, several populations are created using several elite varieties as recipients. These populations may not be the same size; one might be used as the primary mapping population (to give statistical power for detection and good confidence intervals), while the others give an indication as to the robustness of QTLs across elite genetic backgrounds. Results from each set of populations could be pooled and analysed as a NAM population to give further confidence. **b** Half-diallel population structure. The set of populations in (**a**) form one column of this matrix, with the matrix filled by population sizes produced, assuming RILs are used. A QTL identified across donors in one recipient (e.g. Recipient-1 NAM) gives information on diversity of donor alleles, allowing control of false-negative rates in marker design but little control over the false-positive rate; a QTL identified across recipients (e.g. Donor-1 NAM) gives information on diversity of recipient alleles/haplotypes, allowing control of false-positive rates in marker design. The latter is far more important in most cases, so this should be followed if resources are limiting

Even at the current capacity and knowledge level of twenty-first-century molecular biology, it is still very difficult to accurately predict whether a QTL will be effective in other genetic backgrounds. To mitigate these risks, a more direct empirical method is required before QTLs can be deployed for breeding. One approach is to directly map QTLs in multiple backgrounds. Traditional breeding approaches often employed diallel crosses, where one (or more) male lines are crossed to multiple females, precisely to address the issue of phenotypic stability across a range of relevant genetics. Applying this concept to QTL mapping populations, it is evident that a half-diallel cross strategy using diverse elite recipient parents would go a long way towards enriching results for QTL validated across genetic backgrounds (Tsaih et al. 2005; Paulo et al. 2008). In this scenario, a selected donor parent is crossed with multiple elite recipients, with the latter chosen to represent some portion of the genetic diversity of the breeding program. Each of the resulting *F*_1_ plants is used to generate a mapping population; one of these may be a primary mapping population of 200 + lines and the others a reduced size of 100 lines each (Fig. 3). The primary population then gives statistical power to detect QTLs, while the secondary populations give some indication as the generality of those QTLs across the genetic diversity of the breeding program. It should be noted that each set of populations for a given donor then constitutes a NAM design (Yu et al. 2007), and so the results could potentially be pooled and analysed as such to give additional power to reduce QTL intervals. Genotyping costs under current price structures are also nominal. Assuming current genotyping costs between USD $15—$40 per sample to achieve sufficient depth for mapping purposes, spread over a population of 100 lines equates to a project cost around USD $1500—$4000.

Another alternative to constructing and genotyping a number of half-diallel crosses is to proceed directly to deployment of putative QTLs into several elite genomic backgrounds. In this scenario, putative QTLs identified in a primary mapping population (*F*_2:3_, RIL, CSSL, etc.) are backcrossed into several elite varieties—again chosen to represent a measure of genomic diversity in the target breeding program(s)—to produce a series of NILs, which are then analysed for improvement in the trait of interest. Genetically, this is superior as mapping populations only represent a 50% elite genomic background, while NILs would be > 95% and so give a better reflection of the performance of the QTL in the elite backgrounds. It is also likely to be cheaper—even selecting four QTL regions during backcrossing only requires a population size of 145 BC-*F*_1_ plants at each generation (see below; a BC-*F*_1_ population of 145 plants is needed assuming 5 positive progeny are required and a 5% risk of failure is tolerable—the mean number that would be found is approximately 9 positive lines). This is easily achieved in rice and other cereals. The main trade-off with deployment of QTLs is between time and effort—it is more laborious to produce BC_4_*F*_2:3_ families than to produce RILs using rapid generation advance (RGA; Collard et al. 2017). On the other hand, if the QTLs do prove effective, the resulting deployment products can be directly entered into the breeding programs as elite donors for new QTLs are not present among current breeding lines. The NILs produced also represent excellent materials for fine mapping of the target QTLs and validating their effectiveness across environments.

#### Breeding considerations

The third major consideration when determining the relative value of a locus to a breeding program is its effect size. Clearly, if the locus is making minimal difference to a trait, then the return on investment for a breeder to utilise MAS is very low. Such small-effect QTL is much better dealt with using phenotypic or genomic selection (Heffner et al. 2011; Sorrells 2015). In traditional QTL mapping exercises, the percentage of variation explained (PVE, equivalent to the *r*^2^) is often reported, and QTLs are generally considered ‘major effect’ if they show *r*^2^ values over some threshold, typically 20%. This method must be used with caution; the *r*^2^ is a measure of the *variation* explained in that particular mapping population. Depending on the parents (and thus the phenotypic range the population can offer), a QTL can exhibit a high PVE, but may have little biological value. This is often the case with highly inbred populations such as advanced generation backcross progeny (sometimes called backcross introgression lines, BILs) and CSSLs, where the only locus segregating is the target locus, and so the PVE is expected to be almost 100%, but the phenotypic difference may be quite low.

To avoid these biases, it seems better to assess not the percentage of variation explained for the *population*, but the absolute variation explained relative to the elite recipient parent. This is more difficult to extract from QTL mapping software, but could be estimated, for example, as the product of PVE and population variance, or could be estimated by taking mean values for the population divided into genotypic classes for the target QTL. For highly homogenous populations (BILs, CSSLs, QTL deployment products), this is easily estimated as the mean difference from the recipient parent.

As to what effect size justifies deployment of the QTL in a breeding program, this is a value judgement and boils down to a business decision by the organisation consistent with the perceived value of the trait, the cost involved in deploying the QTL, and the frequency of the favourable allele among elite breeding lines. For example, a verified QTL causing an increase in yield potential of 10% would be unquestionably valuable in most breeding programs. By contrast, a 10% improvement in a minor or secondary trait is unlikely to be worthwhile investing a MAS effort. Since this value judgement depends on the value of the trait, the value of the QTL, *and the cost of deploying the QTL*, it is obvious that no matter what the scenario, reducing the costs of deployment will enable more QTLs to be effectively utilised in breeding programs. If the cost of deployment is low enough, it makes sense to conduct deployment during the verification stage as outlined above. Deployment products would then be available to breeding programs immediately with no further investment.

### How can we have confidence in a marker?

Establishing confidence in the ability of a QTL to deliver on the phenotypic variance is relatively well understood by breeders and geneticists alike. But the second and more frequently overlooked correlation that poses reputational risk to MAS as a strategy is between the QTL and the marker used to impose selection for it (QTL ↔ marker). The inability to properly measure and account for this correlation is one of the primary drivers of ambiguous MAS results in modern breeding programs. The performance of the marker systems used to select target QTLs is clearly critical to success, yet strangely there is a shortage of the literature on this subject. Technical performance of a marker is sometimes assessed—notably the proportion of samples giving a result (i.e. call rate), although the consistency of these results between biological replicates is less often assessed. While important, these are usually trivial cases, essentially assessing whether the polymorphism works as a marker in the first place.

In the context of marker-assisted breeding, the far more important consideration is whether the polymorphism is going to accurately select for the target QTL across populations and generations. Unlike through the 1990s and early 2000s when many of the major QTL now known were being identified, the 2010s offer a number of cheap, high-throughput, and highly flexible marker systems. In this modern context, obviously the best-case scenario would be to identify and survey the functional polymorphism itself and productionalise that marker for routine use in breeding. However, the time and expense required to identify the functional polymorphism cannot always be justified by breeding objectives alone, and knowledge of the gene itself is not actually necessary to permit its use in a breeding program. Consequently, most MAS applications in a breeding program identify a marker that is linked to a QTL (usually from the mapping exercise itself) and recommend it as a proxy to select for the QTL itself (e.g. Abasht et al. 2009; Kim et al. 2014). But therein lies the primary reputational risk to marker-assisted selection. Many markers that are physically linked to the QTL and likewise perfectly predict the presence or absence of the QTL in a bi-parental context are not in good linkage disequilibrium (LD) with the QTL once applied to a broader genetic context. Modern breeding programs focused on elite crosses are particularly susceptible to this because LD is a population-level parameter and two linked alleles that are in perfect LD in one gene pool might show little association with another. Thus, markers designed based on success in one breeding program or in one pre-breeding exercise and then applied to others are unlikely to work well, and thus, either the association must be empirically determined for each breeding population, or the number of markers assayed must be very large (Habier et al. 2009). Compounding this problem further, the genetic diversity present in breeding programs is, in many cases, poorly known thus preventing the accurate evaluation of a new marker’s ability to predict QTL[+] and QTL[−] individuals, and phenotypic categorisation of germplasm as tolerant or sensitive often suffers from epistasis (multiple genes affecting the same trait, e.g. disease resistances) and environmental variation.

These difficulties could be mitigated if markers could be designed that gave accurate selection for a target QTL across all the genetic diversity encountered in not just one but across all breeding programs. If such markers were available, they could be used to reliably fingerprint material across generations and breeding programs. To achieve this, measures of the accuracy of a marker across the allelic/haplotypic diversity of interest are required. Key to this is a clear assessment of the false-positive and false-negative rates for any new marker intended to be deployed for a QTL mapped outside the breeding program. In some cases where the favourable QTL haplotype is extremely rare or coming from a wild relative, any marker linked to the QTL is potentially also sufficiently rare that all breeding lines will have one allele and the new donor lines will have the alternate allele. But in many cases, the QTL identified by the mapping exercise comes from germplasm related to the breeding program, and so polymorphisms within the QTL interval may well be present within the breeding program, even if the QTL itself is not. Thus, measuring the false-positive and false-negative rates of any marker to be deployed as a selection target is essential.

**Fig. 4:**
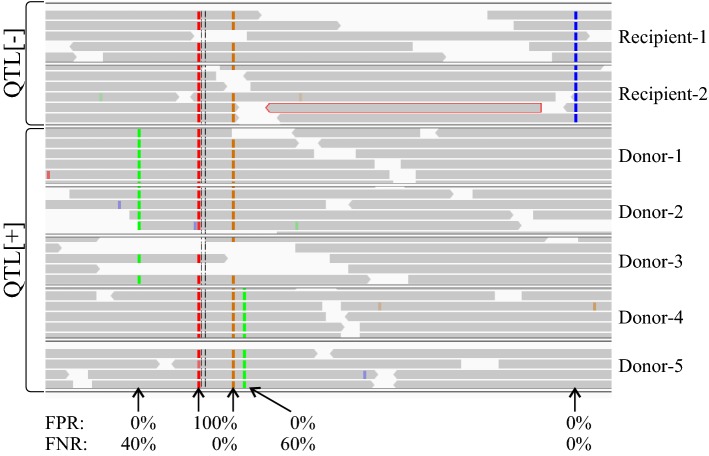
Example of marker accuracy. Single-nucleotide substitution polymorphisms within a particular target gene are shown (coloured vertical lines) across a range of donor and recipient diversity (horizontal tracks) based on whole-genome resequencing data. (Grey bars represent individual reads.) Different polymorphisms show differing levels of association with the target phenotype and thus different accuracy in classifying the known donor and recipient allelic diversity. Clearly the most accurate polymorphism if used as a marker is the last one on the right

Mapping based on the above recommended half-diallel crossing schemes provides an excellent baseline for design of accurate markers, particularly for assessment of false-positive rates. If this mapping structure is followed, by definition several donors and several recipients will be known, thus giving several validated QTL-[+] and [−] genotypic/haplotypic classes. Thus, any marker that classifies some QTL[−] breeding lines as positive is giving false-positive results, while a marker that classifies some QTL[+] lines as negative is giving false-negative results (see Fig. 4).

FPRs are particularly important in the breeding context, as a marker with a FPR > 0% will classify some of the breeding program as positive for the QTL, where in fact it is absent, which results in wasted resources on advancing breeding lines that do not have the intended value, a failure to deploy the QTL among breeding lines in the belief it is already present, or worse, the use of a QTL[−] breeding line as a donor under the false pretence that it has the QTL[+] allele, when in fact it only has the marker allele designated as favourable. The FNR is more likely to be problematic if considering the accuracy of a marker across different breeding programs. In that situation, the allelic diversity found in other programs increases the chance of additional donor alleles being present, which if the marker was too specific to one donor would then be classified as QTL[−].

**Fig. 5:**
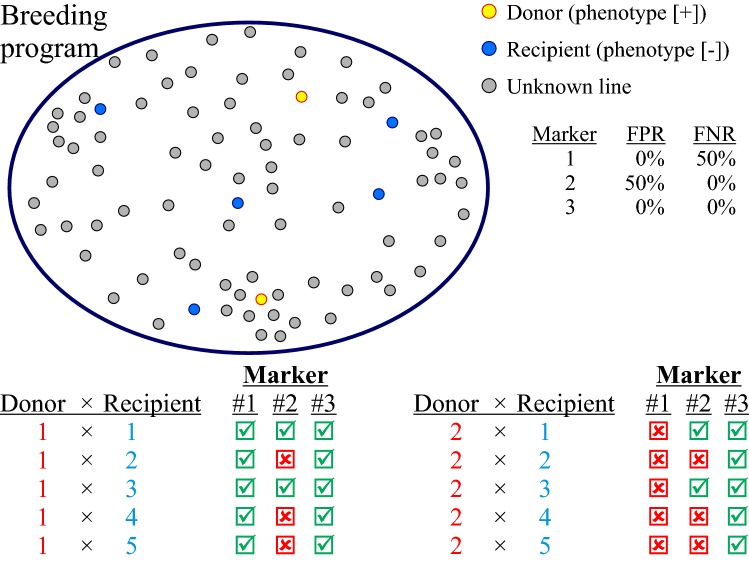
Consequences of selection using accurate versus inaccurate marker systems. Three different markers, all tightly linked with an unknown target gene, are used to select for presence of that gene in several populations. Two alternative donors (with different donor alleles) are crossed with each of five different recipients (all with different alleles). Marker #1 has a FNR of 50%; #2 has a FPR of 50%, and #3 has FPR and FNR of 0%. Marker #1 correctly classifies all recipients as QTL[−]. It is polymorphic with donor #1, so MAS is successful; however, it fails in all populations from donor #2. Marker #2 classifies both donors as QTL[+], but also classifies recipients #2, 4, and 5 as [+], so MAS fails in populations involving those recipients. Only marker #3 correctly classifies all materials as QTL[+] or QTL[−] and so produces reliable marker-assisted selection

An overview of the consequences of using inaccurate marker systems is given in Fig. 5. Utilising advances in genomics, it is now relatively cheap to derive whole-genome sequences for many species, which give a rich source of candidate markers. When combined with a mapping approach designed to characterise some level of donor and recipient allelic diversity, these candidate markers can be assessed for their FPR and FNR to design marker systems accurate across a wide range of allelic diversity and therefore reliable in multiple breeding programs. In the absence of such broad characterisation, it is incumbent upon breeding programs to validate the informativeness of the markers being used to select for valuable QTL before investing expensive MAS resources in increasing their frequency.

## Integrating MAS into a modern breeding system

**Fig. 6:**
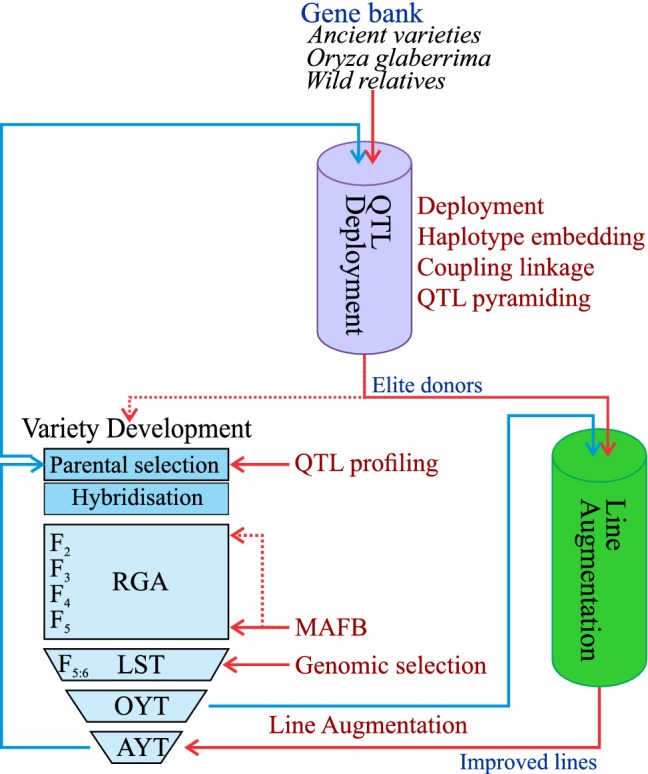
Applications of marker-assisted selection in the breeding process. QTLs controlling traits of interest are identified in upstream pre-breeding processes (trait development) and deployed through QTL deployment. Elite donors from QTL deployment are used directly as parents in forward breeding or used to rapidly increase the frequency of new genes through line augmentation. Variety development processes use marker-aided QTL fingerprints of elite material to inform crossing design and marker-assisted forward breeding to roll out the value of new genes efficiently to new lines developed. Integrated together, these processes can rapidly and efficiently capture the value of loci in the breeding program

Once a breeder is confident in both the ability of the QTL to deliver sufficient phenotypic variance in the breeding program and in the ability of the marker system to accurately track the QTL in breeding populations, the actual implementation of MAS needs to take place. Modern breeding has steadily moved away from the artistic whims of data-poor decisions to an evidence-based, data-driven process, and genotypic information can play a central role in enabling this process. Key issues in the application of MAS revolve around what stage(s) in the breeding cycle MAS should be applied, what population sizes are required to ensure other breeding strategies can be executed effectively, and how MAS integrates with phenotypic and genomic selection methods. For the purposes of this discussion, it will be assumed that the breeding program is using a rapid single seed descent-based line fixation procedure like rapid generation advance (Collard et al. 2017) through to the *F*_5_ generation, and field testing is done on fixed lines. However, similar strategies could be applied if using a pedigree selection approach. Given this approach, marker-assisted selection can be applied at many points within, upstream, and parallel to the forward breeding activities (Fig. 6).

### MAS in forward breeding

If markers of sufficient accuracy are designed, these can be used to derive a fingerprint, or ‘QTL profile’, that articulates the valuable genes and QTLs present in prospective parental lines. This could be used in two ways: to select one parent to contribute an especially valuable gene, or to add value to existing crosses. In a fully modernised breeding program, parents are chosen primarily for their breeding value for quantitative traits, but in many cases, two parents of high potential breeding value in a cross both lack the major QTL of interest. This is common, especially when the QTL is coming from diverse or wild sources and is thus absent or rare in the breeding program. QTL profiling of all potential parents allows for a more informed selection decision to be made between two lines with similar breeding values but differing for the QTL of interest. Similarly, knowing the complete QTL profile of two parents allows for more careful and cross-specific population planning (see below). For example, a cross between two elite parents is not chosen to bring in a particular QTL, but nonetheless each parent may contribute one or more QTLs the other parent lacks. Knowledge of these QTL segregating in a planned population allows the breeder to add value to progeny selected out of the population. If successful, this allows enrichment of the more expensive genomic selection activities and yield trials with material that is known to be QTL positive, thus increasing the value proposition of the yield trials.

This is of course the classical interpretation of marker-assisted selection, and yet despite its familiarity, significant variation exists as to its application in the forward breeding process. It could be applied at early generations (e.g. *F*_2_) or at later fixed generations (*F*_5_ +), and both have distinct advantages depending on the number of genes under selection, the objectives of the cross, and the cost of genotyping. In early generations such as the *F*_2_, the proportion of the population fixed for a gene is only 25%, but a further 50% of the population are heterozygous, allowing for enrichment of a population with a certain favourable allele without constraining all progeny to be homozygous positive. In fixed populations, a much larger proportion is homozygous for the desired allele (46.875% in the *F*_5_, or theoretically 50% for doubled haploids or advanced generation inbreds) allowing for smaller population sizes necessary to arrive at the same number of lines homozygous for the locus under selection. Selection in the fixed (*F*_5_) generation is easily incorporated into any strategy involving RGA, thereby fixing required major loci prior to phenotypic evaluation, and thus field space for phenotypic selection is only used for lines known to possess the required disease resistances and/or quality requirements.

The most critical factor for success of this endeavour (besides accurate marker systems) is to use population sizes sufficient to reliably identify lines positive for the number of genes/QTLs under selection. Determining the population size required can be approximated from the Mendelian segregation ratios, but this will give a consistent underestimate for the required population numbers. For example, assume a breeder wants to select for two unlinked loci and end up with 100 QTL-[+] lines available for the stage 1 yield trial. Given that the Mendelian segregation ratio of a single gene in the *F*_5_ is 0.46875, it is tempting to say that our required genotypic frequency is thus (0.46875)^2^ or 0.2197. Thus, for 100 QTL positive progeny, the population size *n* is given by 0.2197*n* = 100 and thus (rounding to the nearest integer) *n* = 455. However, it must be recalled that under this scenario, 100 positive progeny is the *mean* number that would be observed, not the number that would be observed in any specific population. The actual number observed would follow a distribution centred on this mean (likely a binomial distribution since it is modelling a variable—genotype—with two discrete outcomes—desired and non-desired). Thus, in any given population, there is a 50% chance that the observed number of positive progeny will be below this number. To control for this, an additional term must be introduced: the acceptable risk of failure *F*, being the probability that a population will not yield any positives at all. Then, to calculate the required population size (for a single positive genotype), the following formula can be derived:

Definitions:

*P* = probability of required genotype = segregation ratio

*Q* = number of genes/QTLs under selection

*F* = Failure risk

*R* = required number of positive progeny

*n*(1) = population size required to identify at least one individual with the required genotype

Derivation:

1 $$ {\text{Probability}}\;{\text{of}}\;{\text{positive}}\;{\text{genotype}}\; = \;P^{Q} $$

2 $$ {\text{Probability}}\;{\text{of}}\;not\;{\text{positive}}\;{\text{genotype}}\; = \;1 - P^{Q} $$

Probability of all individuals in population of size *n*(1) having the *non*-positive genotype

3 $$ = \left( {1 - P^{Q} } \right)^{n\left( 1 \right)} $$

The latter equation is then equivalent to the probability of failure, i.e.

4 $$ F = \left( {1 - P^{Q} } \right)^{n\left( 1 \right)} $$

Rearranging this to solve for the population size,

5 $$ n\left( 1 \right) = log_{{\left( {1 - P^{Q} } \right)}} F $$

This then can be used to calculate the population size required to have less than *F* probability of finding *no* positive genotypes. It should be noted that Eq. (5) is conceptually similar to Eq. (4) of Hospital and Charcosset (1997) with selection in a single generation (t = 1 in their formula). However, while the formula from Hospital and Charcosset (1997) is derived for a single QTL genotyped with multiple (flanking) markers over multiple generations, this is derived for multiple unlinked QTLs each genotyped with a single diagnostic marker at a single generation, consistent with the RGA workflow.

Equation (5) gives the minimum population size to have less than *F* probability of not finding *any* individuals with the required genotype. For forward breeding purposes, a single positive individual is not sufficient; each population must give a certain required number of progeny (here designated *R*) for field evaluation. The population size *n*(*R*) required to generate *R* positive progeny could be estimated by assuming *n*(*R*) = *R.n*(1), i.e. it is simply *n*(1) multiplied by *R*. This is, however, an overestimate of the required size; for a population of size *n,* there is also a nonzero probability of finding 2, 3, etc., positive progeny. Taking this into account is far more difficult and requires the use of the binomial cumulative distribution function, where the required number of successes (individuals with the required genotype) = *R*-1, the number of trials is n (population size), the probability of success is the segregation ratio (*P*^*Q*^). This then gives the probability of *not* finding R or more progeny with the required genotype in a population of size *n*. The population size *n* can then be estimated iteratively to achieve the desired probability of failure.

The binomial cumulative distribution of Eq. (5) can then be easily used to calculate the required RGA population sizes for varying numbers of QTLs and required numbers of positive progeny lines for field testing. Examples for up to 5 QTLs and 50, 100, or 200 positive lines for field testing are given in Table 1. It should be noted that, as expected, the required population size for RGA is somewhat larger than if estimated based on the Mendelian calculation, but significantly smaller than if the binomial cumulative distribution is not used in the calculation and it is assumed that *n*(*R*) = *R.n*(1). It is evident that for up to three QTLs, the population sizes required are relatively modest. However, each additional QTL just over doubles the required population size, so beyond three, the required numbers of plants in RGA escalate quickly. If selection for a larger number of genes is required, a modified two-stage selection strategy could be applied. In this strategy, selection is initially applied at the *F*_2_ generation. This would be a disadvantage if selecting for homozygous positive progeny, as the segregation ratio is much smaller (0.25 vs. 0.46875), but if instead heterozygous progeny are included (i.e. eliminating progeny negative for any of the target QTLs), the segregation ratio is actually more favourable (0.75). Subsequent generations will then of course be segregating again for the target QTLs, but the elimination of lines lacking *any* of the targets in the *F*_2_ generation skews the segregation ratio in subsequent generations. Even if no selection is applied, approximately 2/3 of the *F*_5_ generation will be homozygous positive for each target QTL. This means the second round of selection in the fixed generation now has a more favourable segregation ratio (approx. 0.667 vs. 0.46875), and so for a given population size, more QTLs can be fixed with this two-stage selection process. Calculations show that for a single locus, this *F*_2_ enrichment strategy is *less* efficient due to the need to sample twice. For two loci, the number of plants/datapoints is almost equivalent, but for three or more loci, enrichment at the *F*_2_ and subsequent fixation at the *F*_5_ are more efficient; beyond three loci, the savings are substantial.

**Table 1:** Required RGA population sizes for marker-assisted forward breeding

Number of QTLs	Number of fixed-[+] lines required for field evaluation (*R*)		
	50	100	200
1	135	252	480
2	298	555	1050
3	651	1200	2258
4	1396	2580	4840
5	3001	5500	10,350

Required population sizes to yield the specified numbers of fixed-[+] lines for field evaluation, given varying numbers of target QTLs. A fixed acceptable failure rate *F* =  0.01 was assumed, and selection was applied at the *F*_5_ generation (*P* = 0.46875)

### QTL deployment

**Fig. 7:**
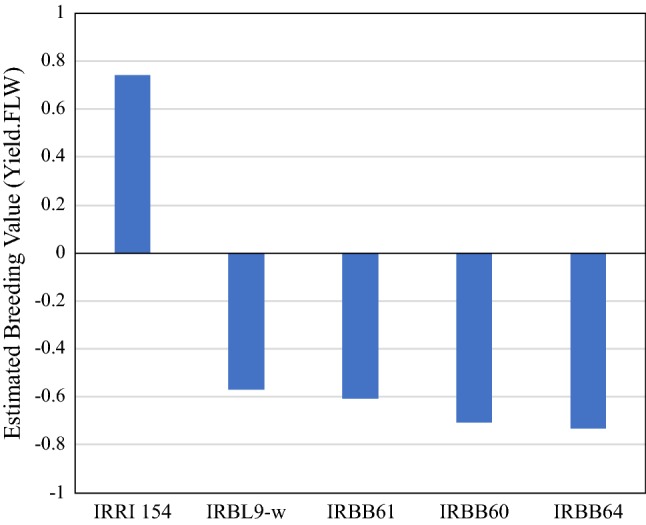
Value of several common donors for disease resistance genes in rice. The breeding value for each line was estimated for yield, corrected for maturity, based on pedigree BLUPs generated using 3 years of historical data from IRRI’s irrigated rice breeding program. IRRI 154 is a high-value elite variety. IRBL9-w is the source of the highly effective blast resistance gene *Pi9*, while the IRBB lines are pyramids of varying combinations of bacterial blight resistance genes *Xa4, xa5, Xa7, xa13,* and *Xa21*

Utilisation of genes in the forward breeding process requires the availability of these genes in elite germplasm, but as shown in Fig. 1, for many genes/QTLs, this is not the case. The desired alleles/haplotypes are often only found in landraces and other germplasm with highly undesirable characteristics. This can be illustrated from the breeding value of several varieties commonly used as donors for disease resistance in rice: the IRBB lines as donors for *Xanthomonas oryzae* resistance genes and IRBL9-w as the donor for the highly effective blast resistance gene *Pi9* (Fig. 7). Clearly, these donor lines could not be used in a breeding program without severely reducing the average performance of resulting progeny. The standard answer to this difficulty is to repeatedly cross the gene(s) of interest into an elite genomic background via marker-assisted backcrossing (MABC), thereby diluting the poor-quality background until its effects are negligible.

The process of taking a gene from a poor-quality donor line and making it available to breeding programs as a high-quality introgression in an elite genomic background is here called QTL deployment. The focus of QTL deployment is on quality of introgressions rather than quantity, with the objective of producing high-quality parents for breeding programs, not necessarily varieties per se. To achieve this, QTL deployment must produce introgressions designed to minimise both genomic penalties of the donor genome and linkage drag around the target gene. These introgressions must be into a recipient that will be relevant to the breeding programs for at least the next 5 years, to enable its use as a parent to introduce the new gene/QTL into the breeding program. Significant value can be added to QTL deployment products if these also take into account haplotypic diversity at the locus of interest to avoid selective sweeps (i.e. embed target genes in multiple elite haplotypes), pyramid multiple genes for a trait, and develop coupling-phase linkages of multiple genes in a genomic region.

Key to producing high-quality introgressions is to reduce risks of linkage drag associated with the target gene or QTL. Backcrossing of the gene of interest into an elite background will dilute the effects of the poor-quality donor genome but without the use of recombinant selection would not remove any negative effects from tightly linked genes. For example, *Pi9* has been observed to cosegregate with unfavourable grain hull colour (Scheuermann and Jia 2016) and has shown a persistent segregation distortion and high levels of floret sterility through multiple heterozygous generations (personal observations). These effects could be due to effects of the *Pi9* gene itself, but are more likely due to linkages with unfavourable genes from the wild progenitor (Amante-Bordeos et al. 1992). Selection of recombinant genotypes, possessing the donor allele at the locus of interest but containing the recipient genotype flanking this region, minimises the risk of linkage drag negatively affecting the quality of the resulting progeny. This recombinant selection requires the screening of a large number of progeny in order to be successful. Population sizes required would vary according to the genetic distance *D* between the peak and recombinant markers. The size required can be easily calculated by incorporating *D* into the segregation ratio of Eq. (5), such that the segregation ratio becomes (*P.D*)^*Q*^, where *D* is expressed in Morgans (so a recombinant at 1 cM is *D* = 0.01):

6 $$ n\left( 1 \right) = log_{{\left( {1 - \left( {P.D} \right)^{Q} } \right)}} F $$

Population sizes obtained from Eq. (6) are quantitatively equivalent to those presented in Table 3 of Hospital (2001) for identical parameters (*F* = 0.01, *t*_2_ = 2, assuming equal population sizes across generations), although the equations presented in the latter publication are unable to solve for a required number of positive progeny (see below).

During backcrossing, the required population size *n* to identify at least one recombinant for a marker at 1 cM with a failure risk of 0.05 is 602, whereas the more favourable segregation ratio in a selfing generation (*F*_2_) means this can be done with only 400 plants. However, since deployment of a gene to the breeding program requires both backcrossing and breaking of linkage drag, conducting recombinant selection during selfing generations adds to the time taken to make a gene useable. Therefore, if it is feasible to generate the population sizes required (as would be possible in a prolific cereal species like rice), conducting recombinant selection *during* the backcrossing process saves 2 generations of time in the deployment process. Similarly, as with marker-assisted forward breeding, it is usually desirable that > 1 positive segregant is identified—in this case to mitigate risks due to mortality of the identified individual. Thus, incorporating Eq. (6) into a binomial distribution as described previously allows the calculation of population sizes for the desired number of positive progeny *R* (for example, if *R* = 2, *D* = 1 cM, *F* = 0.05, the population size required is *n* = 947 BC-*F*_1_ plants).

This first-stage deployment will produce a single, high-quality introgression in one genetic background, and therefore, the newly deployed gene will be available in only one haplotype. This was of course the objective, but if this were used as the sole source of an essential gene in a breeding program, eventually all resulting lines would possess the same haplotype in the region of that gene. The resulting selective sweep would then make a large portion of the surrounding genome unavailable to recombination. If this were only observed at a single locus, its effects would be marginal, but with the number of genes that could be used in rice breeding, it would quickly result in fixation of a substantial fraction of the genome and therefore a reduced genetic diversity in the breeding program and reduced potential for genetic gain.

To avoid this situation, the gene must be deployed into multiple haplotypes. With the cost of sequencing consistently becoming more economical, any modern breeding program should make the effort to evaluate the breeding germplasm for haplotypic diversity. This information permits the breeder to choose three or four elite recipients for this embedding process, each representing a haplotype of some appreciable frequency in the breeding program. If resources permitted, deployment could commence into all of these recipients concurrently. Alternatively, the elite line produced from the primary deployment could be used as the donor for the embedding, in which case only two rounds of MABC-plus-recombinant selection may be required to embed the gene in the second, third, etc., haplotype, since the donor is already an elite background. These haplotype-embedded donors would then be a tremendous resource for quickly rolling out the new gene into an entire breeding program with minimal disruption to the surrounding genetic diversity.

**Fig. 8:**
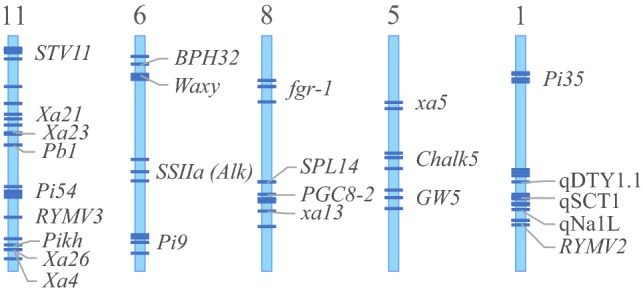
Potential for development of coupling-phase linkages in rice. Several regions of the genome exist that carry multiple major-effect genes in relatively close linkage with each other. However, these genes normally originate from different donors and so are in repulsion-phase linkage. QTL deployment processes can be used to generate recombinant haplotypes bringing the various genes into coupling-phase linkage, easing their use in breeding and increasing the value of the genomic region

Initial deployment of genes is required for any locus of sufficient rarity among elite lines. However, many valuable loci are not completely unlinked, and so even with proper QTL deployment, favourable alleles of most genes would often be found in different varieties, a phenomenon known as repulsion-phase linkage. This can create an ‘either/or’ scenario for the breeder who would need to create inordinately large forward breeding populations to break the repulsion-phase linkage. If careful planning is done up front to account for regions of the genome enriched for potential MAS targets, the QTL deployment process itself can be used to bring these favourable alleles from diverse sources into coupling-phase linkage through harnessing the recombinant selection that is inherent in this process. Coupling of genes in a genomic region adds value to that whole region—selecting for one favourable gene will then typically carry along the other gene(s) simplifying and adding substantial value to breeding selections. Likewise, pyramiding of genes for a trait is well recognised to provide superior phenotypic benefits and stability compared to a single gene; deployment of a new gene can thus be carried out into a variety already possessing one or more other genes for the same trait, thus creating a pyramid as part of the deployment process. The large number of alleles characterised with beneficial effects in rice provides substantial opportunities for these, a few of which are illustrated in Fig. 8.

### Line augmentation

QTL deployment is a laborious and painstaking procedure, requiring detailed oversight of large populations to identify the required recombinants and ensure the quality of products produced. Due to this, it is not well suited to high-throughput applications, and indeed, it only needs to be achieved once into the target elite material—or perhaps a few times, to conduct haplotype embedding. However, this leaves a gap in the utilisation of the target gene in the breeding program; it is now available to crossing programs, but even if all crosses made use of the QTL deployment products as parents, it would still take several years (one complete breeding cycle) for the progeny of these crosses to work their way through to release as varieties or used as a new generation of parents.

Line augmentation is the process to address this lag; it is designed to rapidly introgress genes from QTL deployment products into existing parents, breeding lines, and varieties. These augmented lines can then be used as parents in the current crossing schedule, thereby enabling the deployed gene(s) to have rapid impact and increase in frequency much more quickly. To achieve this, line augmentation involves rapidly backcrossing genes from an *elite* donor into many recipients, utilising only foreground selection. Since only small numbers of backcross progeny are required each generation (Table 2), positive progeny may be maintained through an RGA-like procedure to reduce generation times. As with QTL deployment, the recipient parent is maintained in a staggered planting to ensure synchronised flowering whenever selected progeny mature. To achieve the speed required of augmentation, deployed genes can only be backcrossed two or possibly three times into the recipients, and the volume of lines to be augmented precludes recombinant selection. There is thus no opportunity to correct any linkage drag or poor-quality genomic background in the donor line—the focus of line augmentation therefore is quantity not quality. As such, line augmentation requires high-quality introgressions from QTL deployment to act as donors, ideally as pyramided loci and embedded in the same haplotype as found in the recipient line.

**Table 2:** Required RGA population sizes for line augmentation

Number of QTLs	Number of Het-[+] lines required (*R*)		
	5	10	20
1	20	33	57
2	44	71	121
3	89	147	250
4	182	297	508
5	373	601	1016

Required population sizes to achieve *R* individuals heterozygous for all QTLs, with a fixed failure probability of *F* = 0.01. Segregation ratios for a given locus in BC-*F*_1_ progeny are assumed to be 0.5

Line augmentation can operate in one of two manners. It can be used to deploy new genes into parents of a breeding program, rapidly increasing the frequency of the target genes in many elite backgrounds and thus the benefit of newly identified or previously unutilised loci to be rolled out quickly in new crosses. However, by this method, it would still take one full breeding cycle before the benefits of new genes could be deployed in varieties. Line augmentation can also be used to ‘upgrade’ progeny of existing crosses, perhaps after initial stages of field testing; in parallel with the field testing process, promising lines identified at early stages of testing can be rapidly upgraded within a year with genes they are lacking and upgraded versions inserted back into later stages of field testing the following year. Thus, this ‘upgrading’ mode allows benefits of new genes to be realised even within existing populations and programs, with minimal time lost. Operating together, these two modes (focused on augmenting new crosses and existing populations) could quickly roll out a gene/QTL throughout an entire breeding program.

**Fig. 9:**
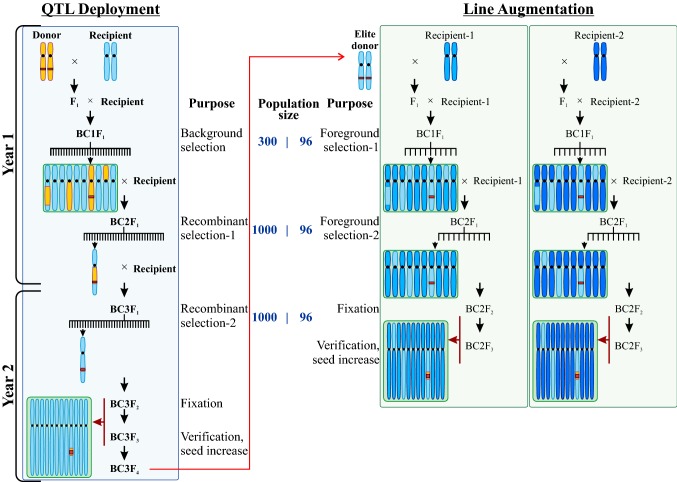
Contrast of workflows between QTL deployment and line augmentation. The focus of QTL deployment on producing quality introgressions necessitates large populations for recombinant selection and advanced backcross generations to clean up the genomic background of poor-quality donor landraces. Line augmentation starts with a high-quality donor from QTL deployment and introduces the new locus into additional elite backgrounds, with only a few backcrosses and no recombinant selection. This means augmentation is faster and far cheaper (per background), but relies on high-quality donor material being available

Since line augmentation requires only foreground selection, it is much less resource-intensive than QTL deployment, yet has a greater impact in terms of lines/breeding material produced (Fig. 9). Therefore, it may be expected that not every breeding program may need to implement QTL deployment activities; it would be more efficient if most could leverage QTL deployment products from a small number of dedicated pre-breeding programs. On the other hand, most breeding programs will benefit from implementing line augmentation at some level to enable rapid conversion of the entire breeding program to new genes.

### Information management

Marker-assisted selection is intimately tied in with management of breeding populations, and ideally, selection decisions based on marker data should be integrated with management of germplasm. Some freely available platforms exist to handle this process, such as the Integrated Breeding Platform (IBP; www.integratedbreeding.net) and KDDart (www.kddart.org); many others are reviewed in Rathore et al. (2018). Often the handling of genotypic data is at a much earlier stage of development than that of handling pedigree, population and phenotypic data, etc., and is typically focused on either graphical overviews of the data (e.g. Flapjack, Milne et al. 2010), calculating recurrent parent recovery rates in MABC programs, or enabling genomic selection, rather than enabling selection of major-effect loci in forward breeding programs. Nonetheless, in many of the preceding use cases, this is adequate for an informed user, particularly if reliable marker systems are available for target genes/QTLs. Where these are available, selection is usually a simple matter of identifying which individuals are positive (or heterozygous) at the target locus/loci and negative at any recombinant or background loci. This process can even be done by simple filter options in standard spreadsheet software, though integration with germplasm management databases is clearly preferable. More advanced usage such as QTL profiling of parental lines and calculation of required population sizes as described do not yet appear to be supported in existing platforms. Proper incorporation of these analytical routines into germplasm management tools is an area that needs further development.

## Strategies for implementing MAS

The preceding discussions have outlined a set of tools and processes that can be used to make effective use of marker-assisted selection in a modernised breeding program. One strategy for integration of these into a coherent breeding strategy is illustrated in Fig. 6. In this model, variety development processes start with the choice of parents, assisted by QTL profiling. Resulting populations are advanced through RGA and progeny with desirable genotypes are identified through marker-assisted forward breeding prior to or during the seed amplification stage (LST); genomic selection would likely happen around the same time. In this way, only those lines carrying the desired favourable QTL haplotypes advance to the more expensive field testing stages. To ensure genetic gain for yield, parents are only chosen from within a defined pool of elite material, and elite progeny are recycled as parents in the next round as rapidly as possible.

QTL deployment acts separately and upstream of this variety development pipeline to deliver new genes in elite genomic backgrounds, which can then be used reliably as parents directly in the breeding program without the risk of negative linkage drag from poor-quality donor genomes. Concurrently, line augmentation operates parallel to the breeding program to roll out new genes from QTL deployment as quickly and widely as possible into material being actively screened by breeders, thereby enabling the conversion of the entire program with a new gene within a few generations. In this way, genetic gains can be maximised through the population improvement approach, without incurring a delayed response to selection as would occur if only one or two elite lines had the new favourable QTL haplotype of interest.

**Fig. 10:**
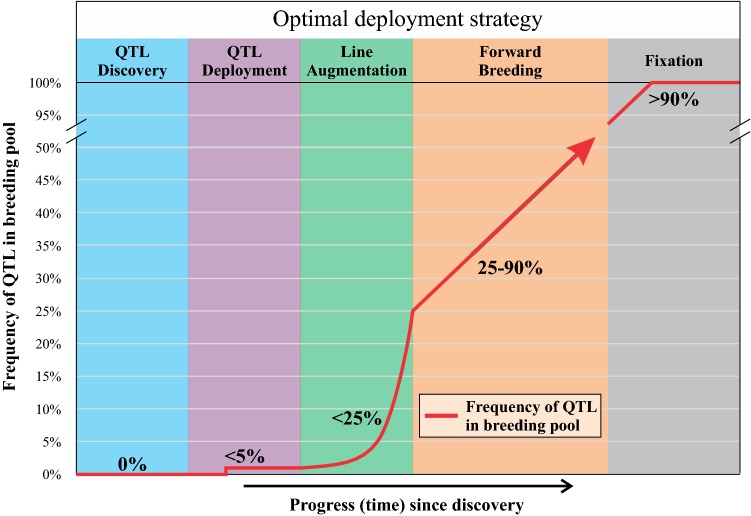
Optimal strategy for using a gene depends on its frequency in elite genetic material. If the gene is initially absent, QTL deployment ensures initial delivery to breeding programs. Subsequently, line augmentation processes rapidly increase the frequency of the new gene in elite genomic backgrounds, to a level where forward breeding can easily utilise the gene

Under this model, the optimal method for capturing value from a MAS target depends on its existing frequency in the breeding program (Fig. 10, Table 3). This can be broken down into three major case studies, based on frequency in the program and value (effect size) of the QTL/gene:

**Table 3:** Optimal deployment strategies, depending on frequency of the target QTL in the breeding program and its effect size (value)

	Effect size (or value)	
	Minor (< 30% ↑)	Major (> 30% ↑)
Frequency in breeding program		
High (> 80%)	Genomic selection	MAS in forward breeding
Moderate (< 80%)	Pyramiding	Haplotype embedding
	Trait introgression	MAS in forward breeding
	Genomic selection	
Low (< 5%)	QTL deployment	QTL deployment
	Pyramiding	Haplotype embedding
	Trait introgression	Pyramiding
	Genomic selection	Trait introgression
		Genomic selection

1. A gene (major or minor effect) is present in the breeding program;
2. A major-effect gene is *NOT* present in the breeding program;
3. A minor-effect gene is not present in the breeding program.

Genes that are already present in the elite breeding program are the simplest case; these can be selected directly through marker-assisted forward breeding once accurate marker systems (as described above) have been established. It matters little whether they have major effects or more minor benefit, as the gene can be selected within existing populations. Examples of such genes in rice would include *BPH32* and the *Wx*^*int*^ allele; these are present at a level where they are likely to be segregating in many elite crosses just by chance, and so no special effort needs to be made to leverage their value other than adjusting the forward breeding population sizes accordingly.

The second case is also simple; it is the case that previous discussions have assumed. A major-effect gene such as *Pi9* or *Xa23* has a very high value and so justifies the effort of QTL deployment and line augmentation processes. These activities then aim to rapidly make the gene available to breeding programs and bring its frequency up to the point where forward breeding processes can make easy use of it.

**Fig. 11:**
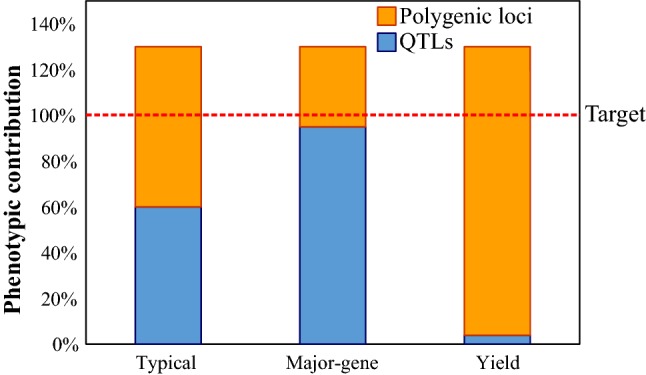
Contributions of significant (detectable) QTLs and polygenic loci to achieving a target trait value under different scenarios of genetic control. Contributions range from a single locus contributing all the desired traits (often seen for disease resistance) to situations where no locus has identifiable contributions (polygenic control; for example, yield). Most examples of new traits fall between these extremes, with a few loci having measurable effect, but to achieve a desired trait value, these must be combined with polygenic loci

The third case remains to be discussed. This is the situation where QTL mapping has failed to identify a single locus controlling a large portion of the trait, but instead has identified potentially three or four moderate-effect loci all contributing in small ways to the trait, either in a synergistic or additive manner. Indeed, this seems to be the general case for many complex traits in rice. It is common for mapping studies to identify three or four loci with measurable effects ranging from 30% down to 10%, and which together control up to 70% of the variation in the population under study (e.g. Famoso et al. 2011; Septiningsih et al. 2011), with the remainder presumably explained by polygenic loci and environmental noise. Individually, these loci may not be valuable enough to go through the full QTL deployment → Line augmentation → Forward breeding process, and yet together they do achieve a large fraction of the desired phenotype (as illustrated in Fig. 11). Therefore, if they were available to the breeding program, they could go a long way towards meeting the desired phenotypic target for relatively little expense, so to ignore these loci seems counter-productive.

This is where a cost–benefit decision must be made. Deployment of these loci as QTLs (without stringent recombinant selection) will have a cost of a few thousand dollars, and if all these loci are from the same donor, it can be achieved in a single backcrossing project. Line augmentation would then be another few hundred dollars per additional variety. Alternatively, if some levels of deployment were incorporated into the trait development activity from the start, there would be no additional cost to utilising these genes in forward breeding processes. The cost of making the QTLs available to the breeding program is therefore modest, yet could significantly enhance breeder’s ability to improve the trait of interest. As such it seems worthwhile for even modest-effect loci to be made available to breeding programs through QTL deployment and line augmentation; they may or may not be selected directly in forward breeding crosses, but if the trait has value, they may still increase in frequency as part of a phenotypic or genomic selection strategy. In this way, the value of even modest-effect loci can be captured in the breeding program even if the effect size does not justify the full deployment of MAS.

## Future possibilities

MAS is thus not a new breeding technology. However, the appropriate application of MAS is still very necessary to a modern breeding system. Further, technological advances and cost reductions in genotyping have allowed for more sophisticated applications of MAS than what would have been possible when breeders were limited to low-throughput marker systems. As MAS has matured over the last decades, it is reasonable to assume that changes are still on the horizon, and as the technological landscape continues to rapidly evolve, so will the applications and strategies associated with capturing the genetic value of novel variation.

One obvious innovation in MAS on the horizon is the advent of gene-editing technologies such a CRISPR-CAS9 (Cong et al. 2013; Mali et al. 2013; Jinek et al. 2013). Putting the discussion of regulatory approval aside, if gene-edited plants were considered ‘generally recognised as safe’ (GRAS), it would allow for an entire breeding program to have a few hundred of the best breeding lines edited to place favourable alleles for all known genes in coupling-phase linkage with no negative linkage drag (Cong et al. 2013; Arora and Narula 2017) and no need for any further forward breeding with markers (as every cross would be fixed for the positive allele). Using a multi-locus allele replacement strategy, QTL deployment and line augmentation could be done is a single step for any major QTL of interest in a few months.

Likewise, many large effect QTLs that could be of potential value to a breeding program are ‘locked’ within exotic or wild germplasm. Often these lines are difficult to evaluate due to shattering seed, prostrate growth habit, unpredictable flowering behaviour, and other characteristics that have been eliminated through domestication (Thomson et al. 2003; Li et al. 2006; Konishi et al. 2006; Sweeney et al. 2006; Zhu et al. 2013), and so the valuable loci may never be identified. However, a ‘domestication’ construct could be created and introduced via gene editing which would permit pre-breeders to more effectively explore wild genetics. Such a construct containing key domestication loci (Zhu et al. 2013; Li et al. 2013; Sun et al. 2017) could reduce the phenotypic noise in mapping populations, enhance the ability to introgress discovered genes/QTL, and reduce the generation time for creating and evaluating populations.

While currently in vogue, gene editing is not the only innovation coming on the horizon. Even if genotyping were free and reliable, logistical factors related to sampling of plants, turnaround time for results, and analytical complexities can be daunting when implementing MAS and integrating it into a variety development workflow. All three concerns would be neatly avoided if genotyping technologies were available to read a genotype directly on individual plants in the field. Such on-plant genotyping technologies that permit real-time, ‘point-of-care’ screening of QTL haplotypes are becoming increasingly possible and economical and are already being applied in medical fields (e.g. Marziliano et al. 2015). Even if only applied to a single major locus at a time, such technologies would allow a breeder to make marker-assisted selections directly in the field as quickly and efficiently as plant height or flowering data are currently collected. This would revolutionise both forward breeding and line augmentation and would cut the costs of QTL deployment in half by eliminating the need to genotype QTL[−] plants.

The efficiency of QTL deployment could also be enhanced greatly by reducing generation times. QTL deployment selections are not well suited to full-scale RGA plant management due to the high value of identified recombinants and the need to produce large backcross progeny from these recombinants. Genetic approaches to reducing generation time could significantly enhance the speed at which new loci could be brought into breeding programs. Such early flowering loci are not favoured in elite material due to yield penalties associated with extreme early maturity, but could be segregated out at the last generation of backcrossing. This would allow new genes to be deployed faster or better-quality products to be developed in the same period of time.

## Conclusion: an integrated model for marker-assisted breeding

**Fig. 12:**
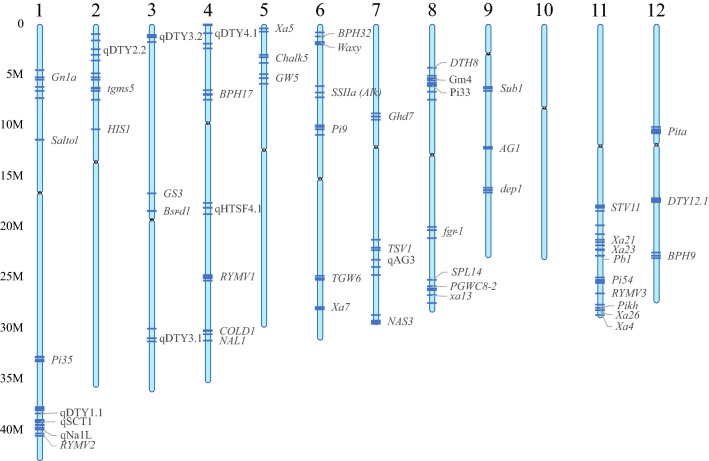
New, accurate SNP marker systems developed in rice. Genomic positions of markers are shown, together with the target QTL. For most genes, both peak and flanking recombinant markers are available. All peak markers are designed to be as accurate as possible based on known donor and recipient material, with false-positive and false-negative rates of 0% wherever possible

As shown in Figs. 1 and 2, despite the large number of QTLs identified and cloned in rice, there remain relatively few that have been used extensively for breeding purposes. This means substantial opportunities exist for the exploitation of these genes in modern breeding programs, and efforts are underway to achieve this. To date, programs attempting to use these genes have almost universally relied on old SSR marker systems. The explosion in genomics resources such as the 3000 rice genomes project (The 3000 Rice Genomes Project 2014; Wang et al. 2018) and low-cost genome resequencing at many service providers (Rasheed et al. 2017) now enables the development of highly accurate SNP marker systems even on a restricted budget. This has been accomplished for over 60 QTL and gene targets in rice (Fig. 12), and these markers are being made available to public rice breeding programs at high-throughput service providers (http://cegsb.icrisat.org/high-throughput-genotyping-project-htpg/).

Likewise, the recognition that few of these genes have made impacts in public rice breeding programs enables breeders and trait developers to address this problem much more actively than in the past. QTL deployment and line augmentation processes are currently underway at the International Rice Research Institute and select national rice breeding programs to upgrade the capacity of breeding programs and enable them to leverage the value of these genes easily and cheaply. Tied in with this, a redesigned concept of trait development is being implemented to ensure QTL mapping efforts produce tangible results in the breeding process and enrich the discovery outputs with QTL useful and relevant to breeding programs. Together these elements are redefining the way QTLs are used in breeding programs consistent with the technological landscape. Instead of being a curiosity, an academic endeavour, or largely ignored, the potential of even modest-effect loci can be efficiently harvested through the integrated breeding approach described in herein. This model thus enables a dynamic and integrated selection approach that accounts for both marker-assisted forward breeding as well as more quantitative population improvement-based approaches in a way that was not achievable 10-15 years ago.

### Author contribution statement

JDP and JNC developed and refined the ideas and protocols presented herein. All authors contributed to the writing and approved the final version of the manuscript.
